# Effects of Light on the Ochratoxigenic Fungi *Aspergillus ochraceus* and *A. carbonarius*

**DOI:** 10.3390/toxins13040251

**Published:** 2021-03-31

**Authors:** Haiyong Zhang, Gang Wang, Qingli Yang, Xu Yang, Yongquan Zheng, Yang Liu, Fuguo Xing

**Affiliations:** 1 College of Food Science and Engineering, Qingdao Agricultural University, Qingdao 266109, China; zhanghaiyongwork@163.com (H.Z.); rice407@163.com (Q.Y.); yangxu1995Y@163.com (X.Y.); 2 Key Laboratory of Agro-Products Quality and Safety Control in Storage and Transport Process, Ministry of Agriculture and Rural Affairs, Institute of Food Science and Technology, Chinese Academy of Agricultural Sciences, Beijing 100193, China; wanggang02@caas.cn; 3 State Key Laboratory for Biology of Plant Diseases and Insect Pests, Institute of Plant Protection, Chinese Academy of Agricultural Sciences, Beijing 100193, China; yqzheng@ippcaas.cn; 4 School of Food Science and Engineering, Foshan University, Foshan 528231, China

**Keywords:** *Aspergillus ochraceus*, *Aspergillus carbonarius*, light, OTA, OTA biosynthesis genes

## Abstract

Ochratoxin A (OTA) usually contaminates agricultural products such as grapes, oatmeal, coffee and spices. Light was reported as an effective strategy to control spoilage fungi and mycotoxins. This research investigated the effects of light with different wavelengths on the growth and the production of OTA in *Aspergillus ochraceus* and *Aspergillus carbonarius*. The results showed that the growth of both fungi were extremely inhibited by UV-B. Short-wavelength (blue, violet) significantly inhibited the production of OTA in both fungi, while the inhibitory effect of white was only demonstrated on *A. ochraceus*. These results were supported by the expression profiles of OTA biosynthetic genes of *A. ochraceus* and *A. carbonarius*. To clarify, the decrease in OTA production is induced by inhibition or degradation; therefore, the degradation of OTA under different wavelengths of light was tested. Under UV-B, the degradation rate of 10 μg/mL OTA standard pure-solution samples could reach 96.50% in 15 days, and the degradation effect of blue light was relatively weak. Furthermore, infection experiments of pears showed that the pathogenicity of both fungi was significantly decreased under UV-B radiation. Thus, these results suggested that light could be used as a potential target for strategies in the prevention and control of ochratoxigenic fungi.

## 1. Introduction

Mycotoxins are small-molecule secondary metabolites produced by microscopic fungi. Crops are susceptible to mycotoxins producing fungi during growth, harvest, storage and transportation. Due to the frequent occurrence of mycotoxins in food and their well-known toxicity, it represents a grave safety hazard. Ochratoxin A (OTA) is a mycotoxin that uses dihydroisocoumarin as a polyketide moiety and is coupled to the amino acid phenylalanine via a peptide bond. It is mainly produced by several species belonging to the genera *Aspergillus* and *Penicillium*. The multiple toxic effects of OTA seriously threaten the health of humans and animals. It was reported that OTA causes human Balcan Endemic Nephropathy (BEN) and has teratogenicity, immunosuppressive, genotoxicity, cytotoxicity and carcinogenicity effects. Some studies also expounded that the carcinogenic effects of OTA were caused by direct and indirect mechanisms, including genotoxicity, oxidative stress and epigenetic factors [1,2,3,4]. Therefore, OTA was classified as a potentially carcinogenic substance (group 2b) by the International Agency for Research on Cancer [5]. OTA contaminates a variety of agricultural products such as grapes, cereals, coffee and spices [6]. When domestic animals are fed with OTA-contaminated feed, it can accumulate in animal and meat products, and the level of contamination is related to the OTA content in the feed [7].

In recent years, some studies have analyzed the effects of environmental factors on the production of mycotoxin; among them, light plays a vital role and it is the key signal that gives living organisms the ability to adapt to the environment. Many biological pathways may be tightly linked with light signaling, such as primary metabolic pathways, the production of secondary metabolites and sporulation [8]. Light has different effects on mycotoxin biosynthesis, depending on light intensity and wavelength, as well as on the species of fungi. In *P. nordicum* and *P. verrucosum,* blue (455–470 nm) and red (627 nm) wavelengths decreased the biosynthesis of OTA by regulating the expression level of ochratoxin polyketide synthase [9]. When *P. verrucosum* and *A. niger* grew under white light, OTA production was suppressed [10]. Compared to incubation under constant darkness, the biosynthesis of OTA by *P. nordicum* was reduced by around 20–30% under constant daylight at a certain intensity [11]. Dunlap and Loros found that circadian rhythms regulated gene expression related to dark/bright changes [12]. In *A. stenyii* and *P. verrucosum*, the high-intensity (1700 lux) royal blue (455 nm) wavelength completely inhibited fungal growth and OTA production [7]. When *P. verrucosum* was exposed to white, blue (455 and 470 nm), red (627 nm), yellow (590 nm) and green (530 nm) light, or left in the dark, only white light and blue light had a significant degradation effect on secondary metabolites. The results showed that blue light was an important part of the spectrum [13] to influence the growth and metabolism of *P. verrucosum*. From these results, we can conclude that light influences fungal growth and metabolite.

Recently, a consensus biosynthetic pathway was identified from *A. ochraceus* [14]. The biosynthesis of OTA begins with the polyketide synthase (PKS, OtaA), synthesize 7-methylmellein, followed by the formation of OTβ oxidized by a cytochrome P450 monooxyme (OtaC). A nonribosomal peptide synthetase (NRPS, OtaB) catalyzed OTβ and a phenylalanine to condense OTB. OTB is chlorinated to OTA by a halogenase (OtaD). These four genes and a transcription factor (OtaR1) are arranged as a cluster in the genome. OTA biosynthetic genes in *A. carbonarius* have also been reported, which is consistent with the gene cluster of *A. ochraceus* [15,16,17]. *A. ochraceus* and *A. carbonarius* are an important OTA-producing *Aspergillus* species. To our knowledge, little is known about the influence of light on these fungi, especially for OTA biosynthesis. There are no scientific data on the impact of light at gene expression level in *A. ochraceus* and *A. carbonarius*. In this paper, the effects of different light wavelengths were studied on the growth of two fungal species, *A. ochraceus* and *A. carbonarius,* and OTA production was also characterized. Additionally, the influence of the light conditions on OTA synthesis was also tested at the molecular level. Our results contribute to a better understanding of the regulatory mechanism of light conditions on *A. ochraceus* and *A. carbonarius*. We propose that a specific wavelength of light is a potential target for the development of strategies to prevent ochratoxigenic fungi infections.

## 2. Results

### 2.1. Effect of Light on Fungal Growth and Morphology

In order to explore the effect of different light wavelengths on the growth of *A. ochraceus* and *A. carbonarius*, the strains were inoculated on the YES medium exposed to different wavelengths of light.

**Figure 1:**
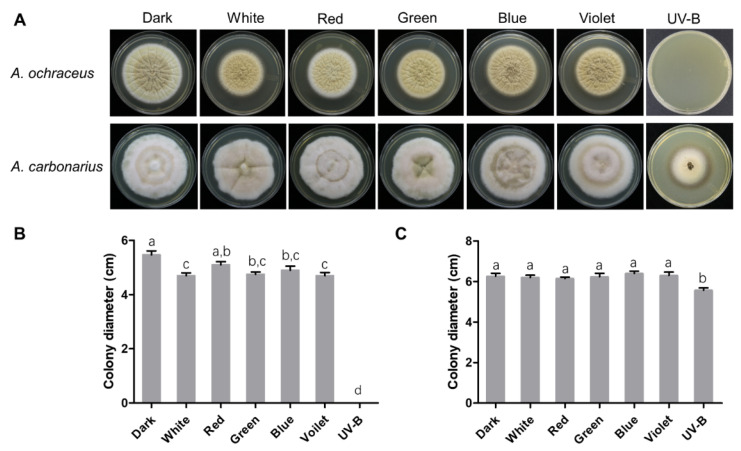
The morphology of *A. ochraceus* and *A. carbonarius* under dark and light conditions. (**A**) A colony view of *A. ochraceus* and *A. carbonarius* under different light wavelengths. (**B**) The colony diameter of the *A. ochraceus* under different light wavelengths. (**C**) The colony diameter of the *A. carbonarius* under different light wavelengths. Each treatment indicates the average of three independent experiments and the bars indicate standard error. Different letters indicate significant differences (*p* < 0.05) between different treatment groups.

As shown in Figure 1A, the colony diameter of *A. ochraceus* on day five was measured. Compared with a dark condition, the growth of *A. ochraceus* was significantly inhibited by white, blue and violet light (Figure 1B), and UV-B light completely inhibited the growth. A deeper pigmentation could be observed when the colonies were cultured under light irradiation (Figure 1A).

The colony growth of *A. carbonarius* was observed and recorded on the sixth day. The colonies exposed to different wavelengths showed a series of concentric circles with a darker hyphae, which was not observed under a dark condition. UV-B inhibited the growth of *A. ochraceus*. No significant difference in colony diameter was observed between dark and other light wavelengths.

### 2.2. Analysis of OTA Production

**Figure 2:**
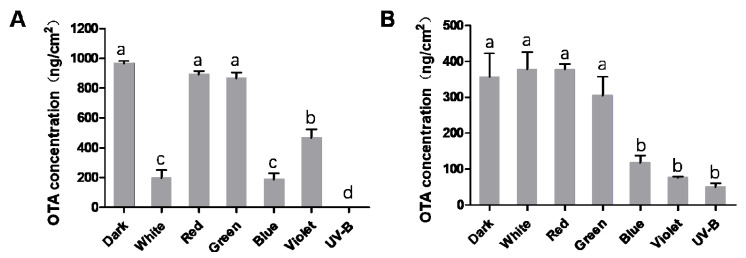
Ochratoxin A (OTA) production of *A. ochraceus* and *A. carbonarius* under different wavelengths. (**A**) OTA production of *A. ochraceus.* (**B**) OTA production of *A. carbonarius.* Each treatment indicates the average of three independent experiments and bars indicate standard error. Different letters indicate significant differences (*p* < 0.05) between different treatment groups.

In order to investigate the effects of different light wavelengths on the biosynthesis of OTA, the OTA production of *A. ochraceus* and *A. carbonarius* under different wavelengths of light were measured by HPLC. The quantitative results showed that blue light, violet light and UV-B had significant inhibitory effects on OTA biosynthesis of two kinds of fungi. *A. ochraceus* produced about 965.94 ng/cm^2^ OTA on YES medium under a dark condition. The OTA contents under white, blue and violet light were reduced by 79.65%, 80.46% and 51.61%, respectively, compared to that under a dark condition. There was no significant difference in OTA production among the conditions of red light, green light and dark for *A. ochraceus* (Figure 2A). *A.carbonarius* produced about 357.05 ng/cm^2^ OTA on YES medium under a dark condition. OTA production decreased by 66.88%, 78.76% and 85.80% under blue light, violet light and UV-B. White, red and green lights have no inhibitory effects in *A. carbonarius* compared with the dark (Figure 2B). Taken together, these results indicated that short-wavelength lights had the potential to inhibit OTA production, among which UV-B had the most obvious inhibitory effect.

### 2.3. OTA Biosynthetic Genes Expression of A. ochraceus and A. carbonarius Were Regulated by Light

Light is a regulatory factor of OTA biosynthesis. In order to further study the regulation of OTA production by different light wavelengths, we detected the expression level of OTA biosynthetic genes. Based on the results of OTA inhibition, we focused on the expression of five OTA biosynthetic genes (*otaA*, *otaB*, *otaC*, *otaD* and *otaR1*) of *A. ochraceus* and *A. carbonarius* under white, blue and violet light on YES medium.

**Figure 3:**
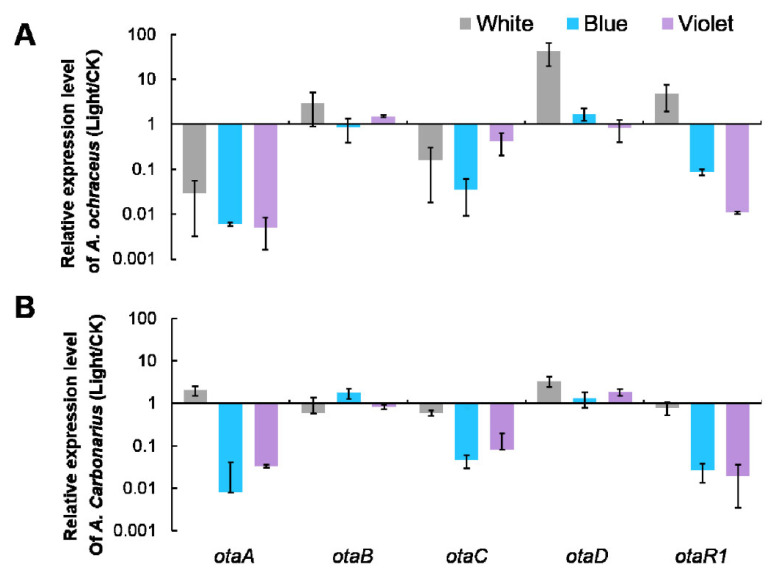
The expression ratio (light/dark) of OTA biosynthetic genes under different wavelengths. (**A**) OTA biosynthetic genes of *A. ochraceus.* (**B**) OTA biosynthetic genes of *A. carbonarius.*

As shown in Figure 3A, the relative expression of the gene *otaA* was significantly down-regulated under three light sources compared with dark, especially under blue light and violet light, where the *otaA* gene was down-regulated more than 100-fold. The expression of the gene *otaB* did not change under the three light sources. The relative expression of *otaC* was down-regulated more than 84.15% and 96.46% under white and blue light, and down-regulated about 57.90% under violet light. The relative expression of the gene *otaD* was up-regulated under white light, and there was no significant difference between blue light and violet light, compared with a dark condition. The relative expression of the gene *otaR1* was down-regulated by 92.42% and 98.92% under blue and violet light, respectively. Combining the above results, OTA biosynthetic genes were down-regulated under OTA suppressing light conditions. White, blue and violet light may decrease OTA production by inhibiting the biosynthetic genes in *A. ochraceus* through direct or indirect pathways.

As shown in Figure 3B, the relative expression of genes *otaA*, *otaC* and *otaR1* was significantly down-regulated under blue and violet light compared with dark. The expression of genes *otaB* and *otaD* did not change under the three light sources. The relative expression of *otaA* was down-regulated more than 99.20% and 96.70% under blue and violet light. The relative expression of the gene *otaR1* was down-regulated by 91.42% and 98.02% under blue and violet light. There was no significant difference between white light and a dark condition, which was consistent with the toxicity results. The results showed that blue and violet light may decrease OTA production by inhibition of the biosynthetic genes in *A. carbonarius* through direct or indirect pathways.

### 2.4. Degradation of OTA by Light

**Figure 4:**
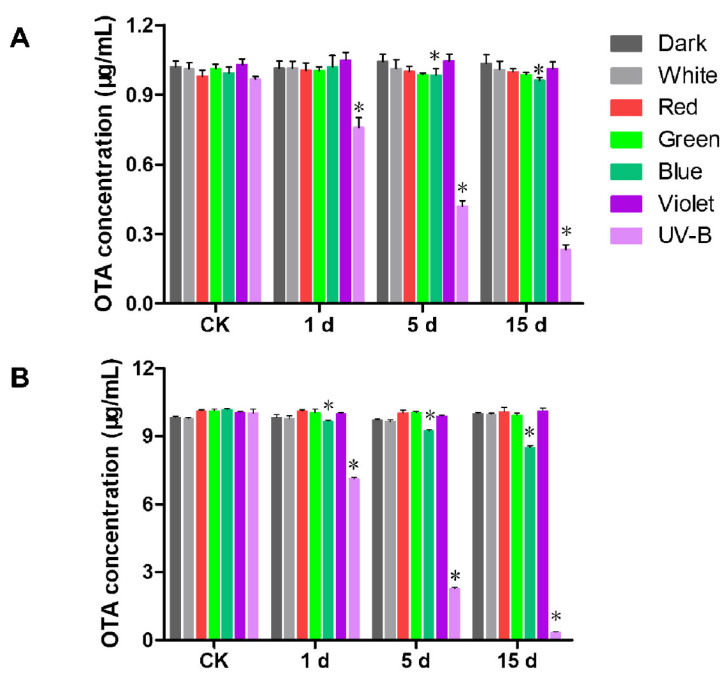
The effect of different light wavelengths on the degradation of OTA. (**A**) The degradation of OTA of 1 μg/mL by light. (**B**) The degradation of OTA of 10 μg/mL by light. Each treatment indicates the average of three independent experiments and bars indicate standard error. Significant differences between different treatment groups are indicated by an asterisk (*p* < 0.05).

To clarify whether light is capable of degrading OTA in the course of decreasing OTA biosynthesis, OTA were treated with different wavelengths of light. The OTA standard pure solution samples of 1 μg/mL and 10 μg/mL concentrations were packed in transparent liquid phase vials and placed in dark, white, red, green, blue, violet light and UV-B, and irradiated continuously at 28 °C for 15 days. As shown in Figure 4, the degradation of OTA standard pure solution samples was not detected under a dark condition. For 1 μg/mL standard pure solution samples, UV-B was able to degrade OTA with a degrading rate of 21.50%, 56.62% and 75.89% at 1, 5 and 15 days, respectively (Figure 4A). Blue light could also degrade OTA statistically, with a degrading rate of 1.33% and 3.52% at 5 and 15 days, respectively. For 10 μg/mL standard pure solution samples, UV-B degraded OTA with the degrading rate of 28.71%, 77.27% and 96.50% at 1, 5 and 15 days, respectively (Figure 4B). Blue light degraded OTA with the degrading rate of 9.32% and 16.46% at 5 and 15 days, respectively. Overall, UV-B and blue light could degrade OTA. It could be confirmed that in a certain range, the higher the concentration of OTA standard pure solution samples, the higher the degrading rate of light under the same conditions and time.

### 2.5. UV-B Inhibited the Pathogenicity of A. ochraceus and A. carbonarius

**Figure 5:**
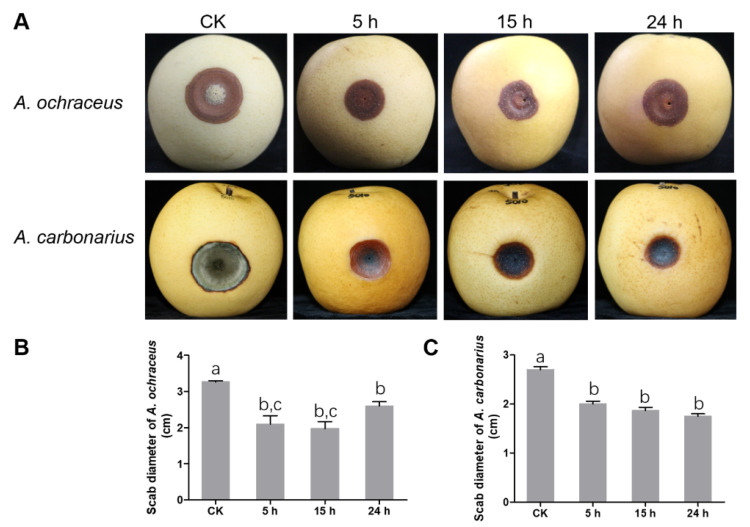
Pathogenicity for *A. ochraceus* and *A. carbonarius* under UV-B irradiation. (**A**) Scab view of *A. ochraceus* and *A. carbonarius* under a dark condition and UV-B irradiation. (**B**) The scab diameter of pears infected by *A. ochraceus* under dark and UV-B irradiation. (**C**) The scab diameter of pears infected by *A. carbonarius* under dark and UV-B irradiation. Each treatment indicates the average of three independent experiments and bars indicate standard error. The different letters indicate significant differences (*p* < 0.05) between different treatment groups.

To further identify whether light has the potential of preventing fungal infection, we explored the effect of different UV-B irradiation time on the infection of pears (*Pyrus bretschneideri Rehd*) by *A. ochraceus* and *A. carbonarius*. The results showed in Figure 5, when UV-B irradiation was used for 5, 15 or 24 hours per day, the scab diameters infected by *A. ochraceus* were about 35.71%, 39.80% and 20.41% less than the infection diameter under a dark condition (Figure 5A). *A. carbonarius* also decreased by 25.90%, 30.74% and 35.18% (Figure 5B). Additionally, a large number of mycelia and spores appeared in the lesion of pears under a dark condition. UV-B radiation significantly inhibited the mycelial growth and spore formation of *A. ochraceus* and *A. carbonarius*. It was interesting that there was no significant difference in the scab diameters on pears with different irradiating times.

## 3. Discussion

OTA is a toxic secondary metabolite produced by the *Aspergillus* and *Penicillium* species. *A. ochraceus* and *A. carbonarius* are major producers of OTA, which contaminates a wide range of hosts. Light controls important physiological and morphological responses in fungi, including development, and primary and secondary metabolism [12,18,19,20,21]. These indicated lights could be developed as a useful strategy to control OTA, and many researchers have made efforts in this area. However, the role of different light wavelengths in regulating ochratoxigenic fungi, especially for *A. ochraceus* and *A. carbonarius*, was not clear until now, and little is known about the genetic regulation of light towards OTA biosynthesis.

In this study, the influence of light wavelengths on growth and the OTA biosynthesis of *A. ochraceus* and *A. carbonarius* was analyzed in detail. The light behavior towards both of the strains was consistent; that is, UV-B possessed the strongest inhibitory effect, while other light wavelengths demonstrated a relatively weak inhibition. These results have also been proven in other fungi [22,23,24]. We found that OTA production of the both fungi was significantly reduced under conditions of continuous exposure to blue, violet light and UV-B, compared to dark conditions. White light inhibited the production of OTA in *A. ochraceus* but not in *A. carbonarius*. Another study showed that white light could inhibit the production of OTA in *A. carbonarius* [8]. This indicates that different light wavelengths might result in great differences in the production of mycotoxins, even in the same fungal species, and that the difference in the results of these experiments is not only due to the light conditions, but also due to other internal or external factors that affect the production of toxins or their interactions.

The expression levels of OTA biosynthetic genes under the different light conditions were also detected to explore the regulatory mechanism of light on OTA production. Under the conditions of blue and violet light, the expression of *otaA*, *otaC* and *otaR1* was positively correlated with OTA production. This indicated that these genes were regulated by light through a direct or indirect pathway. Linking gene expression to light control enables coupling of the entire morphogenetic pathway to light [25]. Fungal photoreceptors and connected signal cascades were described in recent years, and an unexpected complexity has emerged. For example, blue light receptors mainly include LOV-domain-containing proteins, the blue light sensor proteins and the cryptochrome and photolyse protein family. These receptors use down-stream modules such as HOG signaling pathway for signal transduction, and the major response of light is the regulation of gene expression. The relationship between OTA biosynthesis genes and different light wavelengths is close and complex, and further exploration is still needed [26,27,28].

Blue light and UV-B not only inhibited fungal growth and OTA production, but also degraded OTA directly, which demonstrated an excellent potential to control OTA. Therefore, we explored the effect of different UV-B irradiation time on the *A. ochraceus* and *A. carbonarius* infection of pears. The results showed that UV-B radiation had an inhibitory effect on *A. ochraceus* and *A. carbonarius* infection of pears. However, there was no significant difference in the scab diameter of the colonies irradiated at 5, 15 or 24 h per day, which showed that the UV-B radiation of 5 hours per day is sufficient.

We have examined the inhibitory effects of light on both of the fungi, including growth, toxicity, pathogenicity and direct degradation of OTA. In recent years, more than 21 species of fungi were reported to produce OTA, of which more than 16 species could cause different degrees of contamination of agricultural products [29], but *A. ochraceus* and *A. carbonarius* are among the most important fungi. In this research, the concentration of OTA in media, as well as the concentration of OTA directly degraded by light, was higher than that detected in agricultural products contaminated naturally [30,31,32,33]. Therefore, the application of light in prevention and control of OTA needs further research, although we provide the evidence of the fact that light could be a potential target for strategies in the prevention and control of ochratoxigenic fungi.

## 4. Materials and Methods

### 4.1. Solvents and Reagents

Solvents and reagents were obtained from commercial suppliers such as Sigma (Deisenhofen, Germany), Thermo Fisher (Waltham, MA, USA), Oxoid (Basingstoke, UK), Solarbio (Beijing, China) and Tiangen (Beijing, China), and were of the highest purity commercially available.

### 4.2. Strains and Growth Conditions

Two OTA-producing fungi, *A. ochraceus* fc-1 and *A. carbonarius* 5010 were preserved in our laboratory [34,35]. They were inoculated onto YES medium (Yeast Extract 20 g/L; Sucrose 150 g/L; Agar 20 g/L, adjusted to pH = 6.0 with NaOH) for 7 days at 28 °C, after that, spore suspension of each fungal strain was prepared in 0.1% Tween 80. The concentration of spores in the suspension was adjusted to 10^7^ spores/mL, the spore suspension was fully stirred, and 2 μL suspension point was taken and inoculated in the center of the YES medium. The fungal cultures were incubated in the dark and in different light wavelengths. *A. ochraceus* cultures were incubated for at least 10 days, *A. carbonarius* for at least 15 days. At least five inoculated plate cultures were placed in each light condition as technical replicates, and each experiment was repeated three times in the form of biological replicates. The growth rate and OTA production of fungal colonies were detected.

### 4.3. The Light Incubation Conditions

Seven light incubators of different wavelengths were constructed for the incubation of fungi, and darkness as a positive control. Each light incubator was equipped with 5 × 5 W led (Jiangxi Wanjiatong Lighting Technology Co., Ltd, Yichun, China), with the following specifications: A size of 0.5 × 0.6 m. Light conditions were set: incubator 1, darkness; Incubator 2, white light (1867 lux); Incubator 3, red (625 nm, 1895 lux); Incubator 4, green light (470 nm, 1835 lux); Incubator 5, blue light (470 nm, 1815 lux); Incubator 6, violet light (520 nm, 1785 lux); Incubator 7, UV-B (295 nm). These incubators were placed in the same ventilated room with the humidity of 85%. The temperature was measured by placing a thermometer 20 cm perpendicular to the light source to ensure that the fungal colony was incubated at 28 °C. The temperature control of the incubator proved that the heating effect of leds could not be detected within this distance.

### 4.4. Quantification of OTA by HPLC-FLD

To detect the OTA production of *Aspergillus* fungi, *A. ochraceus* was incubated for 9 days and *A. carbonarius* for 8 days at the respective experimental conditions. Six agar plugs about 1 cm diameter from equivalent zones of fungal surface of YES were collected, and extracted with 6 mL methanol (chromatographic level) [36]. After 5 min of vortex, 30 min of ultrasonic vibration, 2 mL of the supernatant solution was filtered into liquid vials with 0.22 μm filter membrane. Next, OTA was detected by HPLC-FLD. In this experiment, the Agilent HPLC system was used to quantitatively detect the OTA content. HPLC-FLD operating conditions as follows: The column was Agilent ZorbaxSB-C18 (4.6 mm × 250 mm, 5 μm); mobile phase was acetonitrile/water/acetic acid (99/99/2, *v/v/v*); flow rate was 1.0 mL/min; injection volume was 20 μL; the excitation and emission wavelengths were 333 nm and 440 nm; operating temperature was 35 °C; each sample was run for 20 min [34].

### 4.5. Isolation of RNA

RNA was extracted using EASYspin Plus Plant RNA Kit of Beijing Lanyi Technology Co., Ltd (Beijing, China) for gene expression experiments, taking the 5 days of *A. ochraceus* and 8 days of *A. carbonarius*, to harvest their mycelium. Mycelium was placed in a mortar and thoroughly ground in the presence of liquid nitrogen. About 100 mg mycelium powder was placed in a 1.5 mL centrifuge tube for RNA extraction. All procedures are essentially the same as those recommended by the kit manufacturer. The integrity of RNA was assessed by gel electrophoresis, and the RNA content was evaluated by the ratio of A260 nm/A280 nm and A260 nm/A230 nm using NanoDrop (Quawell Q5000).

### 4.6. Quantitative Real-Time RT-PCR Reaction and Relative Gene Expression Determination

First-strand cDNA was synthesized using the FastKing RT Kit (Tiangen, China). The relative expression of OTA biosynthesis related genes was monitored by qRT-PCR and carried out using the Sybr Green qPCR Mix (Lanyi, China) with the respective primers. Primers were listed in the Table 1 [14,17,37], and qRT-PCR was performed using the 7500 Real-Time PCR System (Thermo Fisher, USA) with the following cycling program: hold at 94 °C for 3 min, followed by a three-step PCR (40 cycles of denaturation at 94 °C for 15 s, annealing at 55 °C for 20 s, and extension at 72 °C for 31 s). The gene *gadph* of *A. ochraceus* and *β-tubulin* of *A. carbonarius* served as internal standards, and gene expressions were normalized to gene *gadph* and *β-tubulin*. Relative quantification of mRNA expression was established using the 2^−ΔΔCT^ method.

### 4.7. Degradation of OTA Standard Pure Solution Samples under Different Light Wavelengths

For the analysis of different light wavelength degradation of OTA standard pure solution samples, we chose two OTA concentration standard pure solution samples (1 μg/mL and 10 μg/mL) into 2.0 mL transparent liquid vial. The mycotoxins were purchased from Sigma (Deisenhofen, Germany). OTA standard pure solution samples were placed in the dark, white light, red light, green light, blue light, violet light and UV-B, respectively. Incubation conditions were the same as the above two kinds of *Aspergillus*, 28 °C for 15 days. Each light condition was set at three parallels, and each experiment was repeated three times as biological replicates, HPLC-FLD to detect, respectively, 1, 5 and 15 days OTA degradation situation of standard pure solution samples.

### 4.8. The Effect of UV-B on Pathogenicity

The harvested pears were used as a natural medium, *A. ochraceus* and *A. carbonarius* as the infecting fungi. The pears inoculated with the both fungi were irradiated with UV-B for different lengths of time to explore the effect of UV-B on the speed of *A. ochraceus* and *A. carbonarius* infecting pears. The upper surfaces of pears were disinfected three times with 75% alcohol for 10 s; disinfect each time for 10 seconds, and wipe clean with sterile paper. Each pear was punctured by a sterile toothpick into a wound with a depth of 1–2 mm in diameter, and the wound was injected with 1 μL of both fungi spore suspension (10^7^ spores/mL) for inoculation [36]. There are three groups in the experimental group, and UV-B irradiation duration: 5, 15 and 24 h per day. The rest of the day was incubated in the dark under the same conditions as the control group. The above incubation conditions were 28 °C and the relative humidity was 85%. The diameter of the scab was measured after 8 days. Each condition was set three parallels, and each experiment was repeated three times as biological replicates.

### 4.9. Statistical Analysis

Each independent experiment was repeated three times, and each internal experiment selected three parallel samples for data analysis. All statistical analyses were performed by using IBM SPSS statistics version 20 and presented with the means and standard deviation. The statistical significances among three experiments were calculated with ANOVA. The mean values were compared by least significant difference (LSD) and Duncan’s test. *p* < 0.05 was considered statistically significant.

## Figures and Tables

**Table 1:** Primers used for the expression of OTA biosynthetic genes.

**Primers (*A. ochraceus*)**	**Sequence (5′ to 3′)**
*GADPH*-RT-F	CGGCAAGAAGGTTCAGTT
*GADPH*-RT-R	CTCGTTGGTGGTGAAGAC
AF*otaA*-RT-F	GGATCTTTATGACCGAATCAG
AF*otaA*-RT-R	CCTTGACCTGAAGAATGCT
AF*otaB*-RT-F	ATACCACCAGAGCTCCAAA
AF*otaB*-RT-R	GAGATGTTCGGTCTGTTCA
AF*otaC*-RT-F	CTTAATACGGTGGTCTACGA
AF*otaC*-RT-R	GAATGATAGGTCCGTATTTCT
AF*otaD*-RT-F	TATTCCCTAGATACCATATCGG
AF*otaD*-RT-R	GCTTCCTTCTGGTTGTTCA
AF*otaR1*-RT-F	GCTTTCAAATCGAATGATTCC
AF*otaR1*-RT-R	GATCGGTTGGAAGTGTAGAA
**Primers (*A. carbonarius*)**	**Sequence (5′ to 3′)**
*tubβ*-RT-F	CGCATGAACGTCTACTTCAACGAG
*tubβ*-RT-R	AGTTGTTACCAGCACCGGACT
AC*otaA*-RT-F	GTCAAGGTCGGGTGCTACAA
AC*otaA*-RT-R	TCGGAATGATACGCGACTTT
AC*otaB*-RT-F	CTCCACCCATCCTCCCGTTC
AC*otaB*-RT-R	AATCCATGTCCTCACCATCGC
AC*otaC*-RT-F	GTGGTTATCCCGCCCAATAC
ACotaC-RT-R	TGCCAGATTCATCCCGATAC
AC*otaD*-RT-F	GAACGCCAGTAGAGGGACAG
AC*otaD*-RT-R	ATGGAGGTGGTGTTGTTGTG
AC*otaR1*-RT-F	AATGGAACCAGCATTGATCTC
AC*otaR1*-RT-R	GACCCAAGCATTCGCTCTA

## Data Availability

Not applicable. Data is provided in the manuscript.
